# The antioxidant activity of some curcuminoids and chalcones

**DOI:** 10.1007/s10787-016-0264-5

**Published:** 2016-05-17

**Authors:** Münevver Sökmen, M. Akram Khan

**Affiliations:** Department of Chemistry, Faculty of Science, Karadeniz Technical University, 61080 Trabzon, Turkey; Biomedical Research Centre, Sheffield Hallam University, Sheffield, S1 1WB UK

**Keywords:** Curcuminoids, Antioxidant activity, 2,4,6-trihydroxyphenolic chalcone, Ascorbic acid, Diphenylpicrylhydrazyl radical

## Abstract

The antioxidant properties of the synthetic compound (**C1**)–(**C8**), which comprised 7 curcuminoids and a chalcone, were evaluated by two complementary assays, DPPH and β-carotene/linoleic acid. It was found that, in general, the free radical scavenging ability of (**C1**)–(**C8**) was concentration-dependent. Compounds (**C1)** and (**C4)**, which contained (4-OH) phenolic groups, were found to be highly potent antioxidants with higher antioxidant values than BHT suggesting that synthetic curcuminoids are more potent antioxidants than standard antioxidants like BHT. Using β-carotene-linoleic acid assay, only the water-soluble 2, 4,6-trihydroxyphenolic chalcone (**C5**) showed 85.2 % inhibition of the formation of conjugated dienes reflecting on its potent antioxidant activity.

## Introduction

The phenolic compound curcumin (**C1**) (also known as diferuloylmethane; [1,7-bis-(4-hydroxy-3-methoxyphenyl)-1,6-heptadiene-3,5-dione]) is the predominant biologically active component of turmeric, rhizomes of *Curcuma longa* that belongs to the ginger family, *Zingaberaceae*. Curcumin possesses a variety of pharmacological activities and therapeutic properties, and is a potent antioxidant not only in food systems but also in biological systems. Recently curcumin has received considerable attention due to its various pharmacological activities (Huang and Ferraro 1992; Ruby et al. 1995; Priyadarsini et al. 2003). Curcumin and the co-occurring compounds have been extensively investigated for their anti-inflammatory and anticancer activities. Curcumin and curcumin analogues, therefore, represent a novel class of highly selective COX-1 inhibitors and promising candidates for in vivo studies (Handler et al. 2007). Curcumin has been shown to significantly affect the production of TNF. Thus, suppression of TNF by curcumin leads to inhibition of NF-κB and cell proliferation. Using both the in vitro as well as in vivo models of inflammation, various reports in the literature have shown that curcumin inhibits NF-κB in various tissues via different mechanisms, such as, the suppression of IL-1β induced NF-κB activation via inhibition of IκBα phosphorylation, IκBα degradation, p65 phosphorylation and p65 nuclear translocation which result in the down regulation of NF-κB targets including COX-2 and MMp-9 (Shakibaei et al. 2007).

In this endeavour, many curcuminoids have been synthesised and their structure–activity relationship (SAR) has been reported many times over. The mechanism of action of natural as well as synthetic curcuminoids has been predicted through their antioxidant activity.

Reactive oxygen species (ROS) are formed during normal cell aerobic respiration (Gutteridge and Halliwell 2000) and are the main cause of cell damage involved in chronic diseases like diabetes, cancer, cardiovascular and others (Sugamura and Keaney 2011). Reactive oxygen species are also produced by neutrophils which are highly sophisticated cells that actively seek out, ingest and destroy pathogenic microorganisms (Fialkow et al. 2007). To achieve this essential role in host defence, neutrophils deploy a potent antimicrobial arsenal which includes ROS as oxidants. Antioxidants play an important role in neutralising (ROS) and protecting the cells from oxidative damage. Curcumin is an extremely potent lipid soluble antioxidant and has been suggested to act through its pro-oxidant/antioxidant effects, because, formation of ROS by curcumin and curcuminoids correlates with their apoptotic activity on tumour cells (Mishra et al. 2005). The free radical scavenging activity of curcumin can arise either from the phenolic OH group or from the CH_2_ group of the β-diketone moiety. A reactive free radical can undergo electron transfer or abstract H-atom from either of these two sites.

Some functional foods and plants are important sources of exogenous antioxidants, such as vitamins (Vitamin C and E), flavonoids and thiol compounds. It has been recognised that the mechanism of protection from damaging (ROS) by antioxidants depends upon the nature of the antioxidant (Koleva et al. 2002; Ou et al. 2002).

In an on-going work on curcumin, in our laboratories, we have synthesised a number of curcuminoids and have developed assays to assess their anti-inflammatory and antioxidant activities. In this endeavour, here, we report the antioxidant activities of seven curcuminoids (**C1**)–(**C4**), (**C6**)–(**C8**) with considerable structural diversity and the polyphenolic chalcone (**C5**) (Sui et al. 2012; Zhao et al. 2005).

## Experimental section

### Chemistry: materials and method

Melting points were recorded on Stuart SMP3 digital apparatus; IR spectra were recorded on Perkin-Elmer Spectrum 100 FTIR spectrophotometer with a universal ATR sampling accessory; ^1^H NMR spectra were recorded on a Bruker AC 250 MHz and ^13^C NMR spectra were recorded on a Bruker Avance III 400 MHz spectrometer. Mass spectra (MS) were obtained on VG 770E spectrometer operated in EI mode at 70 eV. TLC analyses were done using Merck aluminium coated silica gel sheets, flash chromatography was performed using BDH flash silica gel and the eluents are indicated in parenthesis for each compound. The ^13^C NMR spectral interpretation was done using the numbering system indicated on the structure for curcumin (**C**1) and the generalised structures in tables I and II (Khan et al. 2012).

2.1.1 The compounds *1,7*-*bis*-*(4*-*hydroxy*-*3*-*methoxyphenyl)*-*1,6*-*heptadiene*-*3,5*-*dione* (**C1**), (*1,7*-*Bis(1*-*naphthyl)*-*1,6*-*heptadiene*-*3,5*-*dione* (**C6**), *1,7*-*Bis(5*-*methylidenebutenolide)*-*1,6*-*heptadiene*-*3,5*-*dione* (**C7**) and *1,7*-*Bis(3,4*-*methylenedioxyphenyl)*-*1,6*-*heptadiene*-*3,5*-*dione* (**C8**) were made by the general procedure for curcuminoid synthesis (Pabon 1964) and are reported elsewhere (Khan et al. 2012). The chalcone *(E)*-*1*-*(4*-*methoxyphenyl)*-*3*-*(2,4,6*-*trihydroxyphenyl)prop*-*2*-*en*-*1*-*one* (**C5**) was prepared according to literature method (Sui et al. 2012; Zhao et al. 2005) in 28 % yield as a dark maroon coloured solid, mp 110–112 °C (Lit. m.p. 107–109 °C (Zhao et al. 2005); FTIR (solid) 1645.26 (>C=O), 2400–3600 (broad peak, OH) cm^−1^; ^1^H NMR (250 MHz, d^4^-methanol): δ 3.78 (3H, s, OCH_3_), 5.80–7.70 (m, 6H, Ar–H),7.70 (d, 1H, *J* = 15.7 Hz, >C=CH–CO–), 7.93 (d, 1H, *J* = 15.7 Hz, >CH=C–CO–), 10–12 (broad s, 3H, –OH).

### DPPH assay

This assay spectrophotometrically measures the colour decay of the stable free radical diphenylpicrylhydrazyl (DPPH) by interaction with an antioxidant (Cuendet et al. 1997; Burits and Bucar 2000). Fifty μL of various concentrations of methanolic solution of the sample was added to 5 mL of a 101 µmol methanolic solution of DPPH. After a 30 min incubation period at room temperature, the absorbance was read against a blank at λ517 nm. Inhibition of free radical DPPH in percent (*I* %) was calculated in the following way:

$$I \, \% = \, \left( {A_{\text{blank}} {-} \, A_{\text{sample}} / \, A_{\text{blank}} } \right) \, \times { 1}00$$

where *A*_blank_ is the absorbance of the control reaction (containing all reagents except the test compound), and *A*_sample_ is the absorbance of the test compound. Concentration providing 50 % inhibition (IC_50_) was calculated from the graph by plotting inhibition percentage against sample concentration. Assays were carried out in triplicate. Synthetic antioxidant butylated hydroxytoluene (BHT) was used as positive control.

### β-Carotene-linoleic acid assay

In this assay, antioxidant capacity of the compound is determined by measuring the conjugated dienes produced from linoleic acid oxidation (Dapkevicius et al. 1998). A stock solution of β-carotene-linoleic acid mixture was prepared as follows: 0.5 mg β-carotene was dissolved in 1 mL of chloroform (HPLC grade), and 25 μL linoleic acid and 200 mg Tween 40 were added. The chloroform was completely evaporated using a vacuum evaporator. Distilled water (100 mL) saturated with oxygen (30 min, 100 mL/min.) was added with vigorous shaking. 2.5 mL of this mixture was added to three test tubes, ethanolic solution (350 μL) of the test compound (concentration 2 mg/mL) was added and the emulsion thus produced was incubated for up to 24 h at room temperature. The same procedure was repeated with positive control BHT and a blank. After completion of the incubation period, absorbance of the mixture was taken at λ490 nm. Antioxidant capacities of the synthetic curcuminoids were compared with BHT and blank run under identical conditions.

## Results and discussion

In all the synthetic curcuminoids (**C2**)–(**C4**) and (**C6**)–(**C8**), the heptadiene part has been kept unmodified with the bis-aryl part of curcumin (**C1**) being modified. Compound (**C5**) is an example of a family of compounds known as chalcones, and was chosen for the study because, it has structural similarities to curcumin (**C1**). Curcuminoids have previously been studied for anti-inflammatory and antioxidant activities (Ruby et al. 1995; Portes et al. 2007; Khan and Adams 1995). However, in our compounds (**C1**)–(**C8**) tested for antioxidant activity, a great deal of structural diversity exists that includes phenolic as well as non-phenolic rings at the two ends of heptadiene chain.

**Fig. 1:**
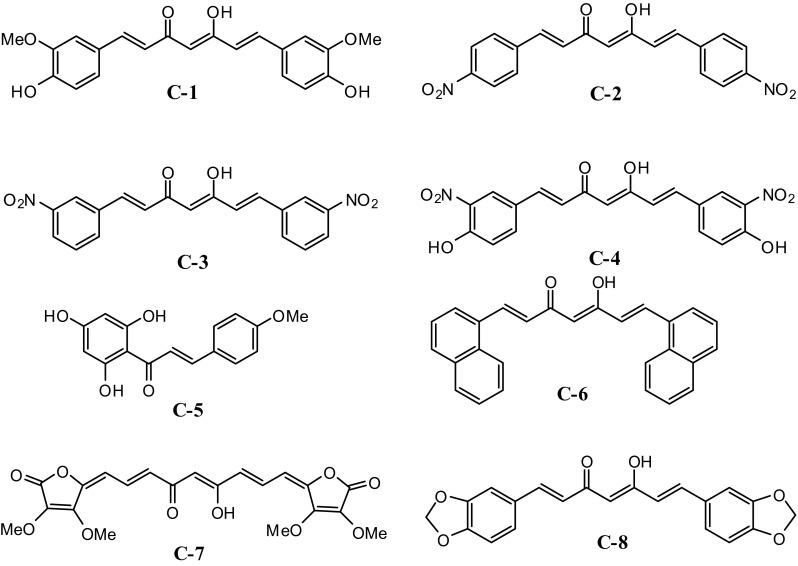
Structures of compounds used in the antioxidant study (Khan et al. 2012; Khan and Adams 1995)

Two complementary assays were employed for screening the antioxidative properties of the synthetic compounds (**C1**)–(**C8**). One of the assays measured the free radical scavenging activity using 2, 2-diphenylhydroxyl stable free radical (DPPH) and a second assay involved the inhibition of the lipid oxidation to determine antioxidant capacity of the samples. The inhibition of linoleic acid oxidation was determined by employing a modified β-carotene/linoleic acid assay (Dapkevicius et al. 1998). In the absence of antioxidants, oxidation products (lipid hydroperoxides, conjugated dienes and volatile by-products) of linoleic acid bleach β-carotene in ethanolic solution. In the presence of antioxidants, oxidation of β-carotene is scavenged, preventing bleaching the colour of β-carotene (Fig. 1).

**Fig. 2:**
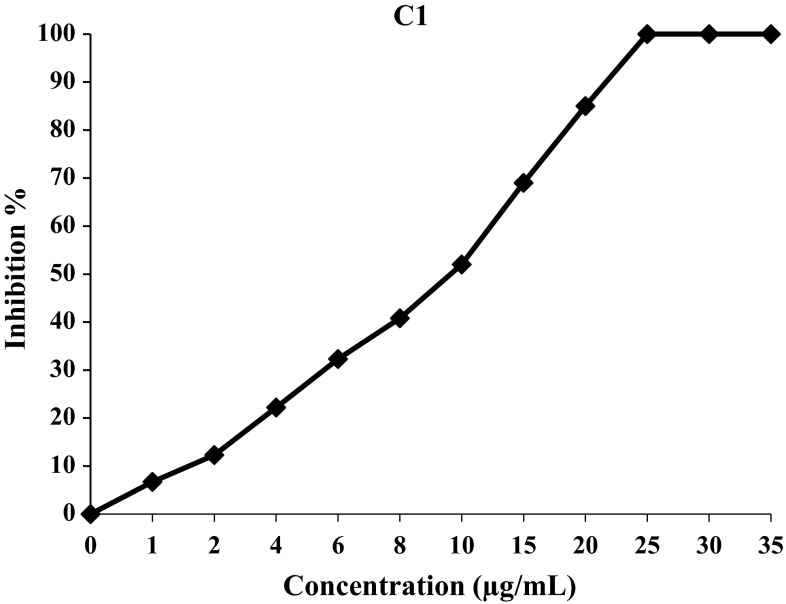
Free radical inhibition percentage of C1 against increasing concentration in DPPH assay

**Fig. 3:**
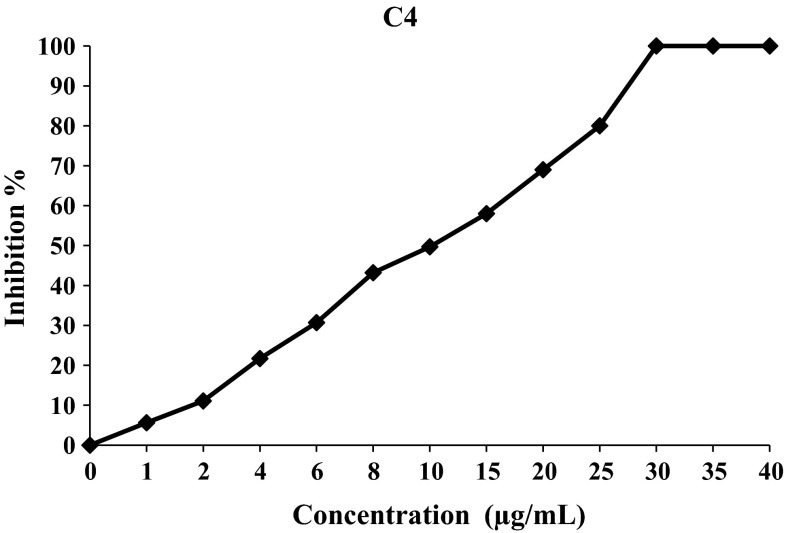
Free radical inhibition percentage of C2 against increasing concentration in DPPH assay

**Fig. 4:**
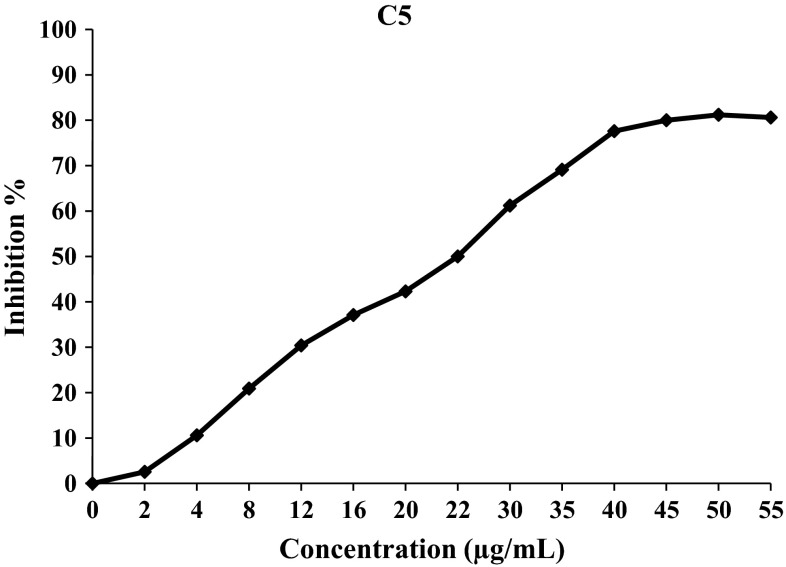
Free radical inhibition percentage of C5 against increasing concentration in DPPH

Using the two assays, DPPH and β-carotene/linoleic acid, to evaluate the antioxidant properties of the synthetic compound (**C1**)–(**C8**), it was found that, in general, the free radical scavenging ability of curcuminoids (**C2**)–(**C4**), (**C6**)–(**C8**) and chalcone (**C5**) was concentration-dependent (Figs. 2, 3, 4).

**Fig. 5:**
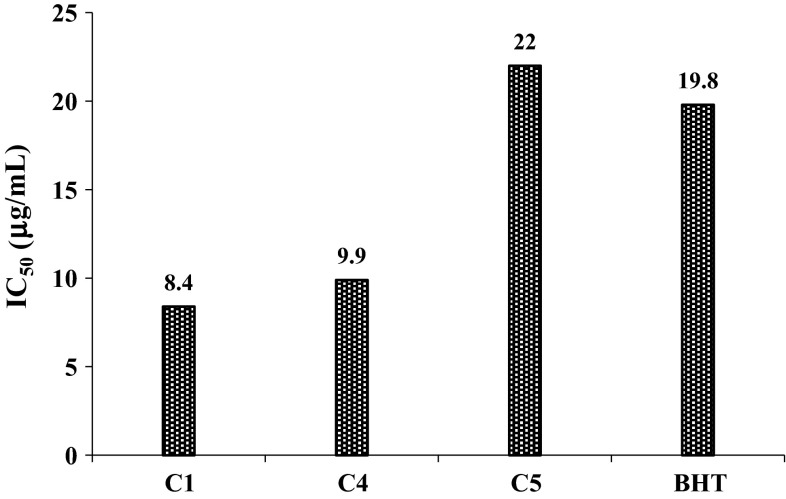
IC_50_ values of active compounds in DPPH assay

**Fig. 6:**
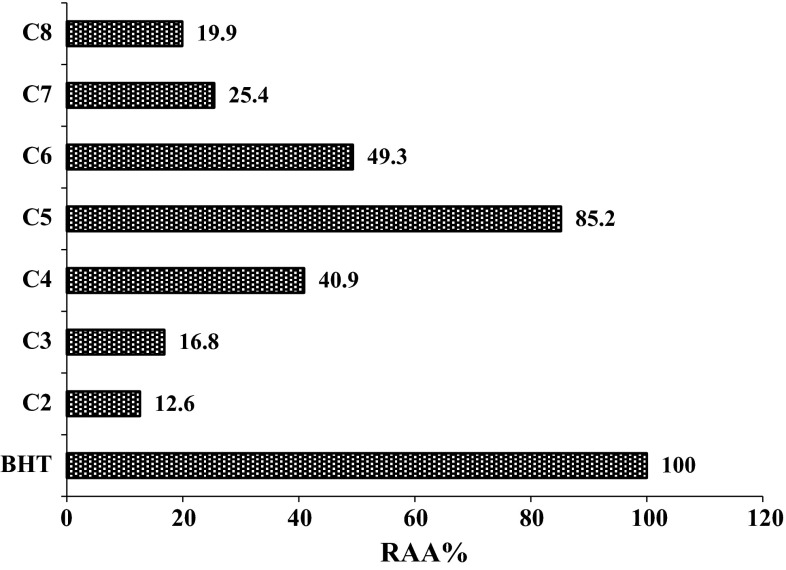
Relative antioxidant activity percentage (RAA %) of synthetic curcuminoids in β-carotene-linoleic acid assay

Free radical scavenging activity of the active compounds (**C1**), (**C4**) and (**C5**) with 50 % inhibition (IC50) was calculated from the inhibition curves and is shown in Fig. 5. The inactive compounds (**C2**), (**C3**), (**C6**), (**C7**) and (**C8**), with no antioxidant or minimum antioxidant activity were not included in Fig. 5. In DPPH assay, the lower IC_50_] was interpreted as higher antioxidant activity of the compound. Compounds (**C1)** and (**C4),** both of which contain a phenolic group at position-4 of the aromatic ring, were found to be highly potent antioxidants with higher antioxidant values than BHT. Thus, synthetic curcuminoids are more potent antioxidants than standard antioxidants like BHT (Fig. 6).

Using β-carotene-linoleic acid assay, only compound (**C5**), which contains phenolic groups at position-4 and 5, showed 85.2 % inhibition of the formation of conjugated dienes reflecting on its potent antioxidant activity. This may be because of two important factors: Firstly, compound (**C5**) is more polyphenolic in nature which augments the antioxidant activity of the molecule. Secondly, the polyphenolic nature of the compound enhances its water solubility, thus amplifying its interaction with linoleic acid present in the emulsion and protecting it from oxidation to yield conjugated dienes. Since the other curcuminoids (**C2**), (**C3**), (**C6**), (**C7**) and (**C8**) did not show significant antioxidant activity by β-carotene-linoleic acid assay, it may be concluded that their interaction with linoleic acid was poor because of their insignificant solubility in aqueous solution. Our results demonstrate that β-carotene-linoleic acid assay is suitable only for water-soluble antioxidants.

**Fig. 7:**
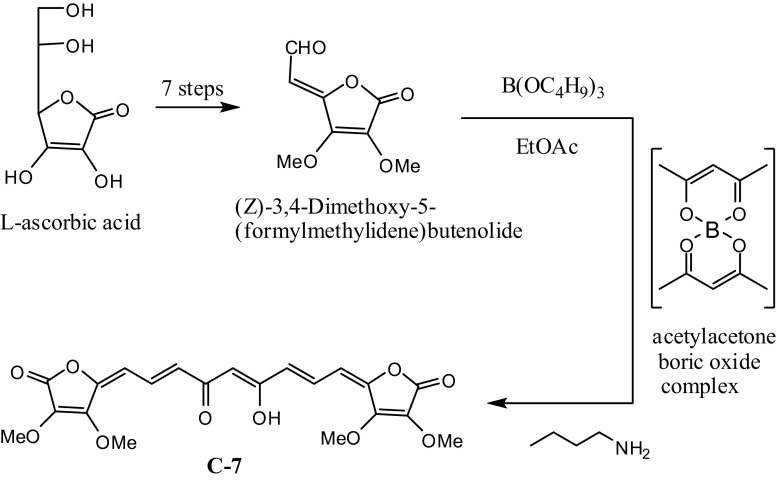
Synthesis of curcuminoid (**C7)** from L-ascorbic acid by the Pabon method

Compound (**C7)**, synthesised in eight steps from L-ascorbic acid (Khan and Adams 1995; Khan et al. 1996; Abaza et al. 2012) (Fig. 7), which showed poor antioxidant activity, is more likely to have good antioxidant activity in vivo, because the methoxy would get cleaved in situ to yield the ene-diol system of ascorbic acid. Likewise, compound (**C8**), which also showed insignificant antioxidant activity, should also be expected to be better antioxidant in vivo due the in situ cleavage of the protected diphenolic system to yield free phenolic groups.

The curcuminoids (**C1**) and (**C6**)–(**C8**), which have previously been studied for anti-inflammatory activity (Khan et al. 2012), and compounds (**C2)–(C4)**, which have been evaluated for their anticancer activity (Hahm et al. 2002) were examined for antioxidant activity. It is known that the mechanism of anticancer and anti-inflammatory activities involves antioxidant activities of the molecules. Thus, the nitro-curcuminoids (**C2**)–(**C4**) and the naphthyl curcuminoid (**C6**), that lack phenolic groups, make interesting candidates for antioxidant study. Our results augment the anticancer activity of these compounds through an antioxidant mechanism.

## Conclusions

It was found that, in general, the free radical scavenging ability of compounds (**C1**)–(**C8**) was concentration-dependent and that antioxidant activity was related to the presence of phenolic groups in *ortho* and/or *para* positions of the aromatic rings in agreement with literature findings. Compounds (**C1)** and (**C4)**, both of which contain the phenolic (4-OH), were found to be highly potent antioxidants with higher antioxidant values than BHT. These compounds offer optimism, as safe candidates, for their possible application in consumer products.
